# Remineralization of enamel caries by an amelogenin-derived peptide and fluoride *in vitro*

**DOI:** 10.1093/rb/rbaa003

**Published:** 2020-03-03

**Authors:** Longjiang Ding, Sili Han, Kun Wang, Sainan Zheng, Wenyue Zheng, Xiu Peng, Yumei Niu, Wei Li, Linglin Zhang

**Affiliations:** State Key Laboratory of Oral Diseases, National Clinical Research Center for Oral Diseases, Department of Cariology and Endodontics, West China Hospital of Stomatology, Sichuan University, Chengdu, China

**Keywords:** enamel, caries, remineralization, amelogenin, peptide, fluoride

## Abstract

Dental caries is one of the most common oral diseases in the world. This study was tantamount to investigate the combinatory effects of an amelogenin-derived peptide (called QP5) and fluoride on the remineralization of artificial enamel caries. The peptide QP5 was synthesized and characterized, and the binding capability of the peptide on hydroxyapatite (HA) and demineralized tooth enamel surface was analysed. Then, the mineralization function of the peptide and fluoride was studied through the spontaneous mineralization testing and remineralization on enamel caries *in vitro*. First, the novel peptide QP5 could bind on the hydroxyapatite and demineralized tooth enamel surfaces. Second, QP5 can transitorily stabilize the formation of amorphous calcium phosphate and direct the transformation into hydroxyapatite crystals alone and in combination with fluoride. In addition, compared to blocks treated by peptide QP5 alone or fluoride, the sample blocks showed significantly higher surface microhardness, lower mineral loss and shallower lesion depth after treatment with a combination of QP5 and fluoride at high or low concentrations. The peptide QP5 could control the crystallization of hydroxyapatite, and combinatory application of peptide QP5 and fluoride had a potential synergistic effect on the remineralization of enamel caries.

## Introduction

Mature tooth enamel is a highly mineralized tissue composed of 95–97% inorganic materials that consists of highly organized nanocrystals of hydroxyapatite (HA) [1]. Dental caries is a major prevalent chronic disease that affects people worldwide. It is caused by cariogenic bacteria that breaks the dynamic re-/demineralization balance [2]. Demineralized enamel is unable to completely repair itself due to its non-regenerative nature [3]. The traditional methods used materials such as silver amalgam, ceramics and composite resin that caused problems involving material aging, metal corrosion and microleakage. Accordingly, the modern dentistry has recently concentrated on developing more effective means to reconstruct enamel to replace the wide use of filling materials, such as remineralization therapies, which try to prevent caries progression and restore tooth mechanical property. As one of the most intriguing processes in nature, biomineralization is essential for the formation of hard tissues such as bone and teeth. Considerable attempts have been made towards the biomimetic synthesis of enamel *in vitro* to repair the lost mineral and restore the structure and function on the surface of enamel [4–8]. Thus, biomimetic remineralization has become an important approach to caries treatment.

Enamel matrix proteins (EMPs) are believed to play a key role in the enamel biomineralization. Amelogenin is one of the most important EMPs in enamel biomineralization that can promote the mineralization and modulate the nanocrystalline structure of calcium phosphate [9]. The crucial amino acids in amelogenin sequence have been identified in previous study which is especially essential for crystal growth. Fan *et al*. reported that amelogenin-releasing hydrogels had the potential for remineralization efficacy using *in vitro* initial enamel caries models [10]. Several biomimetic peptides that contain key amelogenin residues have been shown to have the effect of enamel remineralization [11, 12]. Being one of them, leucine-rich amelogenin peptide has been reported as an alternative material for amelogenin gene expression and an excellent candidate for enamel biomimetic regeneration [13]. However, a few studies focus on the Gln-Pro-X sequence which was highly conserved sequence in amelogenin in different species. In previous research, through searching and analysing the amelogenin sequences in NCBI data bank, we designed an amelogenin-derived peptide QP5 (QPYQPVQPHQPMQPQTKREEVD) comprising five Gln-Pro-X repeats (QPYQPVQPHQPMQPQ) and the C-terminus of amelogenin (TKREEVD), which has shown the ability to promote the remineralization of enamel caries in an *in vitro* pH-cycling system [14]. Meanwhile, our group recently proved that peptide QP5 could promote remineralization in a rat caries model [15].

With decades of clinical success, fluoride has been extensively used for the prevention or treatment of dental caries. Numerous studies have proved the topical fluoride application was effective for the prevention of caries, such as fluoride toothpastes, varnishes, fluoride gels and mouthwashes [16]. It is a known fact that fluoride could enhance mineral uptake and hypermineralization of the lesion surface which would prevent the effective remineralization of the deeper parts of a carious lesion [17, 18]. At the meantime, dental fluorosis induced by excessive fluoride intake as well as the emergence of fluoride-resistant bacteria cannot be ignored [19]. Although when fluoride used properly in the normal individual it presents no problems, the fluoride exposure should be limited among some groups [20]. Thus, there are many investigations focusing on new potential compounds or supplements to promote fluoride remineralization in caries treatment [21]. The combination of fluoride with strontium, calcium phosphate-based compounds, milk, Galla Chinensis and xylitol has been proven to enhance the remineralization effect on dental caries lesions [22–25]. All of these studies have demonstrated that the use of additives or supplements can increase the effectiveness of fluoride for treating caries. However, in the search for alternative materials or complements to fluoride, researchers have turned to the development of bioactive additives that may promote enamel remineralization in a biomimetic way [26, 27]. In view of this study, it was hypothesized that biomimetic peptides might have a combined effect with fluoride, offering a promising new candidate for caries treatment.

This investigation aimed to (i) further explore the biomineralization properties of the novel peptide QP5, and (ii) investigate the combinatorial effects of peptide QP5 and fluoride on the remineralization of artificial enamel caries.

## Materials and methods

### Peptide synthesis and characterization

The peptide on the base of amelogenin was purchased from GL Biochem (Shanghai, China) that consists of residues (QPYQPVQPHQPMQPQTKREEVD). Then it was purified via reverse-phase high-performance liquid chromatography (RP-HPLC) and was characterized by mass spectrometry.

The peptide QP5 was dissolved in 0.1 mM of HEPES buffer to attain a final concentration of 0.2 mg ml^−1^. Circular dichroism (CD) measurements were obtained after incubating at 37°C for 2 and 24 h on a JASCO J-1500 CD spectrometer (JASCO, Tokyo, Japan). The test was recorded in a range of 190–250 nm as the average of five scans using a scanning speed of 1 nm s^−1^ at a bandwidth of 2 nm at room temperature. The data were then analysed by CDPro software (JASCO) and CONTIN/LL was adopted as the algorithm.

### Binding capability of the peptide

#### Binding capability of the peptide on HA

The peptide was dissolved in 10 mM HEPES (pH 7.4) with 5 mg of HA powder (specific surface area of 14.3 m^2^) to yield concentration 20–100 μM in a total volume of 1 ml. After rotating at 37°C overnight, the solution was centrifuged at 14 000 rpm for 10 min to sediment the HA particles suspended in the supernatant. The concentrations of the peptide QP5 in solution before and after incubation were evaluated by a micro BCA protein assay kit (CWBio, Beijing, China) by which we could calculate the amount of peptide bound to the unit surface area of the HA powder. Then linear adsorption isotherms were drawn according to the Langmuir equation (1) [28, 29]. The maximum number of adsorption sites available for the protein per unit of the HA surface area and the affinity of the protein molecules for the HA adsorption sites were obtained from the resulting best-fit line:

(1) $$C_{\mathrm{eq}}/Q=(1/NK)+\;(C_{\mathrm{eq}}/N).$$

*C*_eq_ is the equilibrium protein concentration after incubation with HA (μmol/ml), *Q* is the amount of protein bound to the unit surface area of the HA powder (μmol/m^2^), *N* is the maximum number of adsorption sites per unit of HA surface area (μmol/m^2^) and *K* is the affinity of the protein molecules for the HA adsorption sites (ml/μmol).

#### Binding capability of peptide on the demineralized tooth enamel surface

Extracted human third molars free from caries lesions, cracks and fluoride mottle were obtained and approved by West China Hospital of Stomatology. (It should be noted that the identification of the patients was not made, so the ethical committee approval was not needed for the extracted teeth.) Human third molars were cut into slices and polished to ∼100 mm using water-cooled carbide paper (800, 1000, 1200, 2400, 4000 grit). Enamel slices were demineralized with 37% phosphoric acid for 45 s, rinsed with deionized water, and sonicated for 5 min.

The FITC-labelled peptide was purchased from GL Biochem. Approximately 100 ml of FITC-labelled peptide solution was added to the surface of the normal and demineralized tooth enamel. Then, the coated enamel slides were rinsed three times with deionized water, air dried and visualized using confocal laser scanning microscopy (CLSM, Olympus, Tokyo, Japan).

The peptide powder was examined by FTIR (NICOLET iS10; Thermo Scientific, USA) to obtain a standard spectrum. Then the peptide was dissolved in deionized water to a final concentration of 25 μM. One hundred microlitres of the peptide solution was evenly pipetted on the demineralized tooth enamel surface. After drying at room temperature, each sample was rinsed with deionized water and dried again. ATR-FTIR characterization of the demineralized enamel was recorded before and after peptide coating, and also after deionized water washing.

### Spontaneous mineralization testing

The peptide solution was mixed with solutions of CaCl_2_ and NaH_2_PO_4_ (pH 7.4) to a final concentration of 1.6 mM Na_2_HPO_4_, 3.3 mM CaCl_2_ and 200 μM peptide with or without 500 ppm NaF. For analysis using a conventional transmission electron microscopy (TEM), 10 μl aliquots were dropped onto a carbon-coated Cu TEM grid after 30 min, 2h and 24 h incubation [30]. The same concentration solution in the absence of peptides was the negative control. The characteristics of the crystals in the presence of peptides and fluorine were tested using TEM with selected area electron diffraction (SAED) and energy-dispersive X-ray spectroscopy (EDXS). The TEM images were obtained using a FEI Tecnai F20 S-TWIN electron microscope at 120 kV.

### Remineralization of tooth enamel caries

#### Sample preparation

The crowns of human third molars were cut into sections using a diamond-coated band saw with continuous water cooling (Struers Minitom, Struers, Copenhagen, Denmark). The surfaces were then ground flat with water-cooled silicon carbide paper of various grits (800, 1000, 1200, 2400, 4000 grit; Struers). The enamel blocks were embedded in polymethylmethacrylate and painted with two layers of acid-resistant nail varnish, leaving a 4 × 4 mm window exposed. Prior to caries lesion formation, baseline surface microhardness (SMH) of the enamel blocks called SMH_0_ was obtained using a microhardness tester (Duramin-1/-2, Struers) and a Vickers indenter at a load of 25 g for 15 s. Then, 120 enamel blocks were chosen for further experiment.

#### Carious lesion formation

Enamel initial caries lesions were produced in the demineralization solution contained 50 mM acetic acid (pH 4.5), 2.2 mM Ca(NO_3_)_2_, 2.2 mM KH_2_PO_4_, 5.0 mM NaN_3_ and 0.5 ppm NaF at 37°C for 3 days under continuous low-speed magnetic stirring (100 rpm). Post-demineralization SMH_1_ was measured in an identical manner as before demineralization. The final sample of a total of 60 enamel blocks was selected for further testing.

#### pH-cycling regime

All 60 blocks were subjected to a standard pH cycling per previously reported protocols [31] and randomly allocated to six study groups (*n* = 10 per group): the LF group: 500 ppm NaF; the HF group: 1000 ppm NaF as positive controls; the negative control group: HEPES group; the QP5 group: 25 μM QP5 alone; the QP5-LF group: 25 μM QP5 and 500 ppm NaF; and the QP5-HF group: 25 μM QP5 and 1000 ppm NaF. Enamel samples were immersed in the treatment solution of six study groups four times daily (5 min each time) at 08:00, 09:00, 15:00 and 16:00. After each treatment, the samples were rinsed with distilled and deionized water. In addition, there was a 2 h acid challenge in demineralization solution of 50 mM acetic acid (pH 4.5), 2.2 mM Ca(NO_3_)_2_, 2.2 mM KH_2_PO_4_ and 1.0 mM NaN_3_ from 11:00–13:00 h. The rest of time, samples were immersed in remineralization solution of 20 mM HEPES (pH 7.0), 0.9 mM KH_2_PO_4_, 1.5 mM CaCl_2_, 130 mM KCl and 1.0 mM NaN_3_. The cycle was repeated for 12 days in sealed containers maintained at 37°C with continuous low-speed magnetic stirring (100 rpm). All solutions were prepared fresh daily.

#### SEM–EDXS analysis

After the pH cycling, SEM (Inspect F50; FEI, USA, 20 kV) was used to examine the microstructures of the enamel samples surface. The enamel blocks were sonicated for 10 min, rinsed with deionized water, air dried and sputtered with Au before observation. The chemical component of the remineralized samples surfaces was measured using EDXS (INCA350, Oxford, UK).

#### SMH analysis

The SMH of the enamel blocks after pH cycling (SMH_2_) was measured. The percentage surface microhardness recovery (SMHR) was calculated by the SMH_0_, SMH_1_ and SMH_2_ according to the following equation: SMHR% = (SMH_2—_SMH_1_)/(SMH_1—_SMH_0_) × 100% [32].

#### Transverse microradiography analysis

The enamel sections were carefully cut and polished to ∼120 µm thick which was verified by a digital magnescale indicator (SONY, Tokyo, Japan). The slices were fixed on a Plexiglass slide in a sample holder and microradiographed alongside an aluminium calibration step wedge using a monochromatic CuK X-ray source (Philips, Eindhoven, the Netherlands) operated at 20 kV and 20 mA for 25 s. The lesion depth, mineral loss and mineral content in the different enamel layers were obtained according to the images using Transversal Microradiography Software 2006 (Inspektor Research Systems, Amsterdam, the Netherlands). A software programme was used to calculate the mineral loss and lesion depth of the lesion by comparing the lesion to sound tissue using a step wedge scale. The mineral loss results were reported with units of vol% μm [33]. Lesion depth was defined as the distance from the enamel surface to the point at which mineral content reached 87% that of sound enamel [34]. Mineral content in the different enamel layers was calculated by lesion depth and mineral loss.

### Statistical analysis

All of the statistical analyses were performed using SPSS 17.0 (IBM, Armonk, NY, USA) and plotted using GraphPad Prism software (San Diego, CA, USA). The normal distribution of the data was tested using the Shapiro–Wilk test. Inter-group differences in SMHR%, lesion depth, mineral loss and average mineral content at different depths were assessed for statistical significance using analysis of variance followed by the Student–Newman–Keuls test. To determine whether the peptide in combination with fluoride significantly influenced the observed results, all four treatment conditions were analysed using factorial ANOVA with a 2 × 2 factorial design. The threshold of significance was set at *P* < 0.05.

## Results

### Characterizations of QP5

The HPLC analysis (Supplementary Fig. S1a) demonstrated that the characteristic peak of the peptide was at 10.238 min and its purity was more than 92% according to the ratio of the area of the main peak to the area of the hybrid peak. Meanwhile, the calculation result of MS the mass spectrum of the QP5 peptide was 2660.6 which was close to the theoretical molecular weight 2600.91 (Supplementary Fig. S1b). The result of CD measurement showed that QP5 had a 40.4% of β-sheet/β-turn. Meanwhile, the CD spectrum had no obvious change after 24 h (Supplementary Fig. S2).

### Adsorption capability of peptide on HA and demineralized tooth enamel

**Figure 1:**
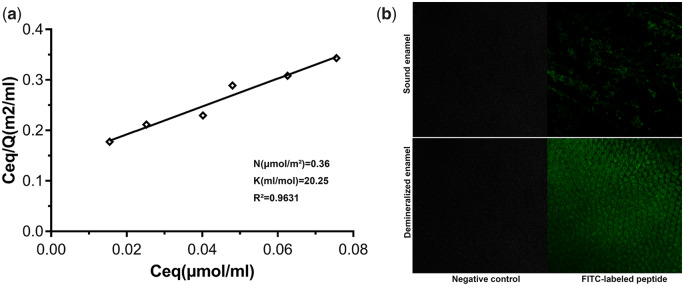
(**a**) Linear adsorption isotherms of QP5. The maxim number of adsorption sites per unit of HA surface area (*N*) and the affinity of peptide molecules for HA adsorption sites (*K*) and were calculated. *R*^2^ is the correlation coefficient obtained for linear adsorption isotherms. (**b**) CLSM images of sound tooth enamel surface, non-treated demineralized tooth enamel sample surface, FITC-labelled QP5-treated normal tooth enamel surface and FITC-labelled QP5-treated demineralized tooth enamel surface

The adsorption isotherms of QP5 on HA fit the Langmuir model well (*R*^2^ > 0.96; Fig. 1a). The binding affinity of QP5 for the HA adsorption sites was 20.25 ml/μmol and the maximum number of adsorption sites per m^2^ of HA available for binding to QP5 was 0.36 μmol/m^2^.

Figure 1b shows the fluorescent dispersion on the enamel sample surfaces tested by CLSM. Obviously, there was no fluorescence on the control enamel sample surface and non-treated demineralized enamel sample surface. The green fluorescence was distributed sporadically on the sound tooth enamel surface after treatment. However, the fluorescence on the FITC-QP5-treated demineralized sample surface was so strong that the enamel prism was also clearly visible. Through the above different methods, we confirmed that QP5 has a good adsorption capacity for tooth enamel and HA, which was conducive to its remineralization function.

**Figure 2:**
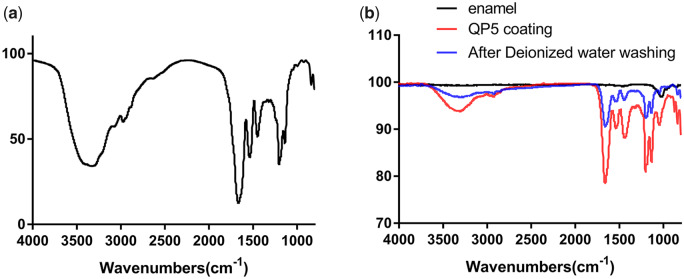
FTIR spectrum of the QP5 peptide (**a**), ATR-FTIR spectra of the demineralized enamel before and after QP5 peptide coating, and also after deionized water rinsing (**b**)

Figure 2a displayed the FTIR spectrum of the QP5 peptide. The peaks from 3500 cm^−1–3100^ cm^−1^ were ascribed to amide N–H stretching, and the peak at 2970 cm^−1^ was contributed by –CH2 vibration. The bands at 1660, 1540 and 1450 cm^−1^ were attributed to amide I, amide II and amide III groups, respectively. The bands at 1200 and 1140 cm^−1^ were due to peaks of ${\text{PO}}_{4}^{3}$^−^. Figure 2b exhibited the ATR-FTIR data for the demineralized enamel samples before QP5 peptide coating, after QP5 peptide coating and after deionized water rinsing. As can be seen, the characteristic peak of the demineralized enamel (1050 cm^−1^ for ${\text{PO}}_{4}^{3}$^−^) can be clearly observed. Then, after QP5 peptide coating, the characteristic peaks of QP5 peptide (3500–3100 cm^−1^ for amide N–H stretching, 2930 cm^−1^ for –CH2 vibration, 1660 cm^−1^ for amide I, 1540 cm^−1^ for amide II, 1450 cm^−1^ for amide III, 1200–1050 for ${\text{PO}}_{4}^{3}$^−^) were obviously detected due to a lot of QP5 peptide reserved on the demineralized enamel surface. After deionized water rinsing, the characteristic peaks of the QP5 peptide were only somewhat weaker than before washing and still apparent indicating that most of the QP5 peptides remained on the surface.

### Spontaneous mineralization testing

**Figure 3:**
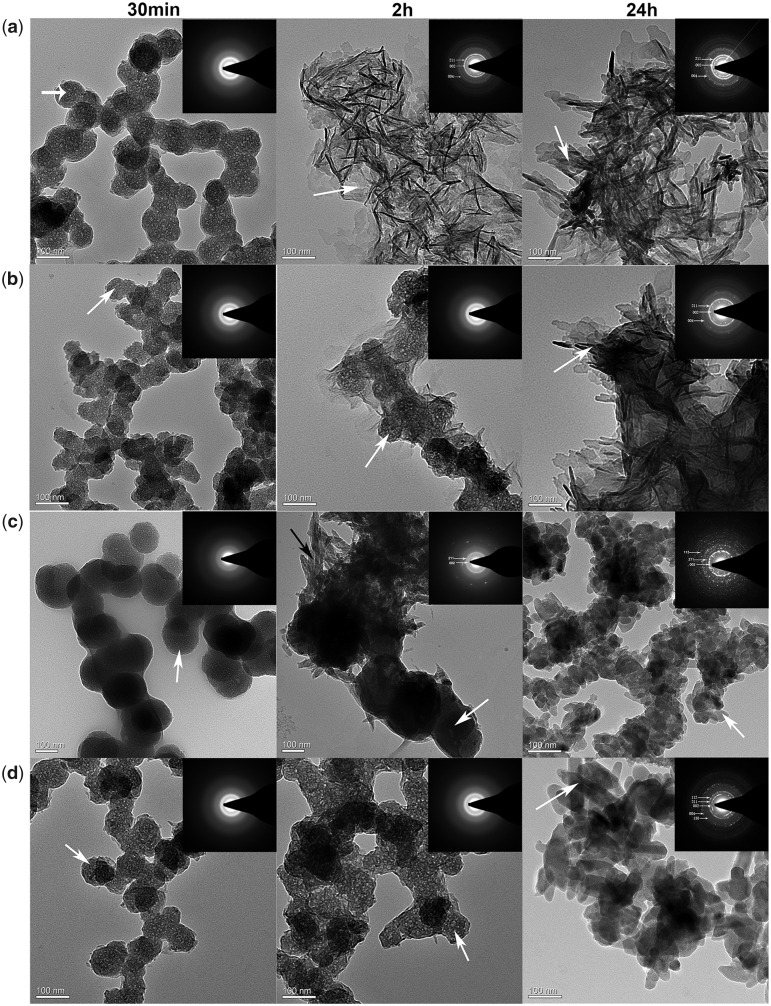
TEM and SAED (insets) images of calcium phosphate minerals formed in the control group (**a**), QP5 group (**b**), NaF group (**c**) and QP5 + NaF group (**d**)

The effects of the synthesized peptides and fluoride on the nucleation and growth of calcium phosphate were analysed by TEM after 30 min, 2 and 24 h of incubation at 37°C (Fig. 3). Meanwhile, the elemental compositional of crystals formed in the four groups after 24 h were analysed by EDXS test. After 30 min, spherical particles (white arrow) were observed in the control group (Fig. 3a; 30 min), QP5 group (Fig. 3b; 30 min), NaF group (Fig. 3c; 30 min) and QP5-NaF group (Fig. 3d; 30 min) that were consistent with the known morphology of amorphous calcium phosphate (ACP) as reported in other studies [30]. The amorphous nature of these particles was confirmed by the broad diffuse SAED ring pattern (Fig. 3; 30 min; inset). In the presence of QP5 with or without NaF, the spherical ACP particles (white arrow) were observed even after 2 h revealed by TEM and SAED analyses (Fig. 3b and d; 2 h). However, randomly plate-like (white arrow) crystals were found instead of spherical particles in the control group after 2 and 24 h (Fig. 3a; 2 and 24 h). SAED analyses showed the diffraction planes of (002), (211) and (004) (Fig. 3a; 2 and 24 h; inset) which were indexed as the hydroxyapatite planes that suggested it formed hydroxyapatite crystals. In the presence of NaF, small rod-like crystals (black arrow) began to form on the surface of the particles after 2 h and all the crystals transformed into short rod-like (white arrow) after 24 h (Fig. 3c; 2 and 24 h). SEAD showed the diffraction planes corresponding to the (002), (211) which confirmed these crystals were made of apatite. In addition, the Ca/P ratio of crystals was 1.58 and the Ca/F ratio of crystals was 3.65 by EDXS test, which was consistent with fluorohydroxyapatite (FHA) minerals in a previous study [35]. After 24 h, longer and thicker plate-like crystals (white arrow) were found in QP5 group comparing with the control group (Fig. 3b; 24 h). SAED analyses exhibited crystal diffraction patterns that were indexed as the HA (002), (211) and (004) planes along the *c*-axis (Fig. 3b; 24 h; inset). The Ca/P ratio of crystals formed in the QP5 group was 1.67, which approximated the theoretical Ca/P ratio for HA in natural tooth enamel. In the presence of QP5 and NaF, elongated rod-like crystals (white arrow) were observed after 24 h (Fig. 3d; 24 h). SAED analyses showed the diffraction planes of (002), (112), (211) and (130), and (004) (Fig. 3d; 24 h; inset) which suggested it formed HA and FHA crystals confirmed by a Ca/P ratio 1.87 and a Ca/F ratio 3.91. The diffraction planes of (002) and (004) indicated that the crystals were aligned parallel in the *c*-axis direction.

### Remineralization of enamel caries *in vitro*

In the spontaneous mineralization experiment in solution, the QP5 peptide and fluoride group showed better nucleation ability and crystal morphology. Therefore, we further constructed the initial enamel caries model *in vitro* to explore the remineralization ability of peptide through the pH-cycling regime.

**Figure 4:**
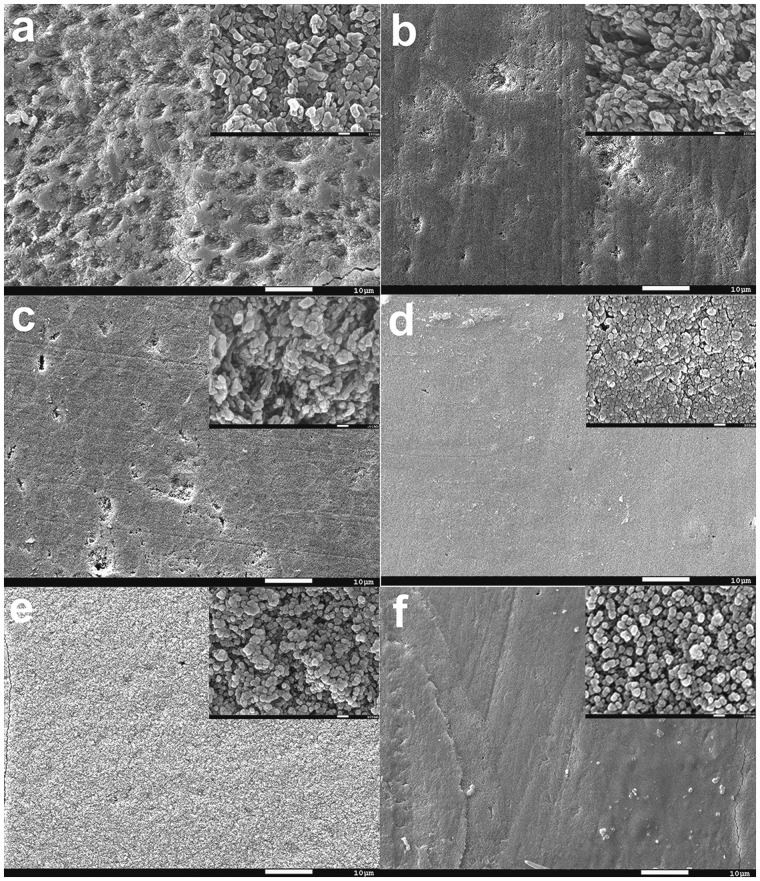
SEM images of the surface of the tooth enamel. Demineralized enamel treated with (**a**) HEPES, (**b**) QP5, (**c**) LF, (d) HF, (**e**) QP5-LF and (**f**) QP5-HF coating after 12-day pH cycling

Figure 4 showed the surface morphology of the tooth enamel samples after a 12-day pH-cycling regime by SEM. Elemental compositional analysis of the surface by EDXS was shown in Supplementary Fig. S3. Regenerated crystals could be observed in all of the groups that show differences in the shape, size, growth direction and distribution on the tooth enamel surface. As shown in Fig. 4a, the renewed crystals in the control group surface were fragmentary and the enamel prism can be seen roughly, which was different from the other groups. Elemental analysis gave a ratio of Ca/P 1.55, possibly indicating a mixed mineral composition of HA and calcium–phosphate transition phase (Supplementary Fig. S3a). On the QP5 peptide treatment group, many rod-like crystals formed on the enamel surface after remineralization, and a ratio of Ca/P 1.63 which was close to ideal ionic ratio of 1.67 in HA (Fig. 4b and Supplementary Fig. S3b). The addition of fluoride induced a transition of rod-like crystals to spherical crystals (Fig. 4c and d). In the LF group, the Ca/F ratio was 6.18 and the Ca/P ratio was 1.45 (Supplementary Fig. S3c). While in HF group, the Ca/F ratio was 3.81 and the Ca/P ratio was 1.52 (Supplementary Fig. S3d). The elemental analysis indicated that the crystals were possibly consisted of CaF2 and FHA. In the presence of QP5 and fluoride, dense spherical crystals covered all of the tooth enamel surface (Fig. 4e and f). Elemental analysis of the samples in QP5-LF and QPF-HF groups gave a ratio of Ca/F 3.96, 3.42 and Ca/P 1.48, 1.46, respectively. (Supplementary Fig. S3e and f). The results indicated the mineral sediments were possibly HA, FHA and CaF2.

**Figure 5:**
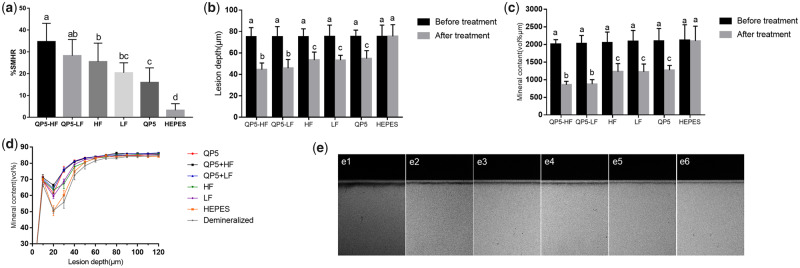
(**a**) Percentage of SMHR after 12-day pH cycling. SD bars labelled with different letters show statistically significant difference, *P* < 0.05. (**b**) Lesion depth (µm), (**c**) mineral content (vol% µm) and (**d**) mineral content (vol% µm) versus different enamel depths (µm) of lesions (c) after 12-day pH cycling. Profiles are average % (± SD) for 10 enamel blocks per treatment group (5 scans per block). (**e**) TMR images of all treatment groups after pH cycling. Bars labelled with different letters are significant differences (*P* < 0.05)

Figure 5a shows that the SMHR% of the NaF groups, the QP5 group and the combination of the NaF and QP5 groups was significantly higher than the HEPES group. With the addition of QP5, the SMHR% of the NaF groups increased obviously in both the HF and LF groups. The greatest SMHR% was observed in the enamel samples treated with QP5-HF, which was significantly higher than in samples treated with either NaF or QP5 alone. The QP5-HF group showed slightly higher SMHR% compared to the QP5-LF group, but the difference between them was not significant. The same results were found between the HF and LF, the QP5-HF and QP5-LF groups.

After pH cycling, the thickness of radiolucent tissue in enamel blocks was tested by transverse microradiography (TMR) (Fig. 5e). The thickness of the radiolucent tissue was thickest in the HEPES group and thinnest in the QP5-LF and QP5-HF groups. The radiolucent tissue was of intermediate thickness in the blocks treated with NaF or QP5 alone. Consistent with these results, the enamel blocks treated with HEPES showed the greatest mineral loss and deepest carious lesions after pH cycling (Fig. 5b and c). Treatment with NaF alone or peptide alone led to similar mineral loss and final lesion depth, whereas the combination of NaF and peptide led to significantly less mineral loss and shallower lesions compared to treatment with NaF or peptide alone. The mineral content at different enamel depths for the six groups is shown in Fig. 5d. The enamel blocks treated with NaF or peptide alone showed similar mineral content that was significantly higher than the HEPES-treated blocks. Importantly, the enamel blocks treated with the combination of NaF and peptide showed significantly more mineral content than all of the other groups, while there was no significant difference between QP5-HF and QP5-LF. However, these five groups showed significantly greater mineral content compared to the samples treated with HEPES (*P* < 0.05). To determine whether the interaction between the fluoride and a biomimetic peptide led to the combination effect of the fluoride and biomimetic peptide on the remineralization of the initial enamel caries, we analysed the treatment conditions using factorial ANOVA with a 2 × 2 factorial design. The results indicated that there was an interaction between the fluoride and the biomimetic peptide when the initial caries was treated with the fluoride combined with biomimetic peptide (*P* < 0.05).

## Discussion

Tooth enamel was a hard tissue consisting of hydroxyapatite that preserved the tooth. More and more researchers were focused on reconstructing enamel through organic–inorganic interactions. Synthetic pH-induced amphipathic self-assembling polymers which could direct mineralization of hydroxyapatite and a biomimetic peptide based on human phosphophoryn capable of nucleating hydroxyapatite which could be used in the repair and regeneration of dental tissue had been reported [36, 37]. Furthermore, it was reported that nanocomplexes of phosphorylated chitosan and ACP promoted the biomimetic remineralization of demineralized enamel [38]. In response to environmental triggers, an oligomeric β-sheet-forming peptide spontaneously assembles into biomimetic scaffolds that nucleate hydroxyapatite crystal formation in enamel [39]. The peptide 8DSS derived from human dentine phosphoprotein had been shown to promote the uniform deposition of nanocrystalline calcium phosphate over demineralized enamel surfaces [40]. Inspired by EMPs during enamel biomineralization, amelogenin hydrogen, peptides containing functional domains of amelogenin such as leucine-rich amelogenin peptide and other synthetic peptides, had been used to promote biomimetic mineralization [11, 41–43]. In previous studies, we synthesized a peptide QP5 derived from amelogenin which promoted enamel caries remineralization *in vitro* and promoted remineralization in a rat model of enamel caries *in vivo*. Furthermore, the peptide in chitosan hydrogel could promote the remineralization of initial enamel carious lesions in a biofilm model [44].

Previous studies have shown that a strong affinity with the substratum was part of the most important characteristics the material should have for enamel remineralization [45, 46]. The acidic amino acids could regulate the orientation of HA crystals when they adsorbed on certain surfaces [47]. Therefore, we believe that the binding property of peptides is critical to promoting enamel biomimetic remineralization. Depending on the Langmuir adsorption isotherm experiment, the binding affinity of QP5 for the HA adsorption sites was 20.25 ml/μmol elucidating that the peptide could bind on the HA. Furthermore, the FITC-QP5-treated demineralized sample was fully covered with fluorescence in CLSM test and the characteristic peaks of QP5 peptide on the treated demineralized enamel samples in ATR-IFTR test indicating that QP5 had been adsorbed on the enamel surface. The peptide QP5 comprised lots of Gln residues in QPX repeated fragment which played an important role in the adsorption of the peptide the HA surface. For Gln residues, the adsorption phenomenon was motivated by the intermolecular H-bonds between the N-containing groups and the phosphate on the hydroxyapatite surface [48]. In addition, some research had proved that the neutral C = 0 groups in Pro residues were associated with Ca^2+^ binding by charge neutralization. In a word, the peptide QP5 could adsorb on the enamel surface.

Some recent studies had revealed that ACP played a key role in the formation of oriented apatite crystals of tooth enamel [49, 50]. As we know, during the development process of mineralized tissues some proteins including acidic amino acids played an important role in controlling the nucleation sites and transforming ACP into the ordered apatite crystals [51]. Furthermore, it was proposed that some analogues of proteins, such as polysaccharide macromolecules and polyamino acid mimicking the functional domain of these proteins, could be used to stabilize calcium and phosphate ions [52–54]. The peptide QP5 comprised the hydrophilic C-terminal domain of amelogenin which included acidic amino acids. According to the TEM results, the peptide QP5 was found to transitorily stabilize ACP formation and finally convert it into oriented hydroxyapatite crystals, and the Ca/P ratio of the crystals was 1.67, approximating the theoretical Ca/P ratio for HA in natural tooth enamel. In the presence of NaF, QP5 could also transitorily stabilize the amorphous nanoparticle and guide it into the elongated rod-like crystals. Some researchers had proved that recombinant amelogenin could briefly stabilize ACP and induce the formation of well-organized bundles of HA, which was dependent on the hydrophilic C-terminal domain [55]. The C-terminal domain of amelogenin had been indicated that it was crucial to the formation of chain-like structures, parallel arrays of apatitic crystals and the lack of this segment caused abnormal mineralization [56–58]. By interacting with calcium ions in ACP, it could transitorily stabilize ACP and guide it into oriented hydroxyapatite crystals.

In the remineralization experiment, a lot of crystals on the enamel surface were induced by QP5, which differs from the control group. The demineralization protocol used in this study is one of the chemical methods which are commonly used in artificial caries model. The main advantage of this method is that the effects of a single factor can be studied by changing the physical and chemical properties of the solution or substrates under highly controlled conditions to provide information on the dynamics of demineralization and remineralization of the surface and subsurface hard tissues of teeth. This method is simple, easy to operate, repeatable and the experiment period is short. It can be used to compare the ability of different organic acids to cause tooth demineralization, to study the de-/remineralization process of caries and the mechanism of fluoride in remineralization [59, 60]. Although this method oversimplifies the pathogenesis of caries and ignores the role of bacteria, saliva and carbohydrate in caries, it is still the most common method in the study of fluoride and natural drugs in preventing caries.

The SMHR% of the QP5 group which was similar to the NaF groups was significantly higher than the HEPES group. The TMR results showed that shallower lesion depth and less mineral loss were found after pH cycling in blocks treated with peptide or NaF alone, while there was no significant change in samples treated with HEPES. All of above results demonstrated that the peptide or NaF when applied alone achieved significant remineralization effects during cycling, suggesting that this peptide promoted enamel caries remineralization with an effect similar to NaF. In the presence of QP5 and fluoride, however, oriented rod-like crystals were observed after 24 h in solution, and dense spherical crystals covered all of the tooth enamel surface. Tooth enamel blocks treated with the combination of NaF and peptide showed significantly greater SMHR% and mineral content than enamel blocks treated with NaF alone. Combination treatment was also related to significantly less mineral loss and shallower lesions after pH cycling than the treatment with NaF alone, suggesting that this peptide does enhance fluoride-promoted enamel remineralization. However, we found that there was no significant difference between the 500 and 1000 ppm fluoride groups, which was consistent with several studies [61]. We speculate that, with 500 ppm fluoride, rapid mineral precipitation covered the lesion surface, the surface pores access to the lesion interior had been blocked, so the concentration increase made no difference. Anyway, the combination of QP5 and NaF could enhance the effect of fluoride obviously.

**Figure 6:**
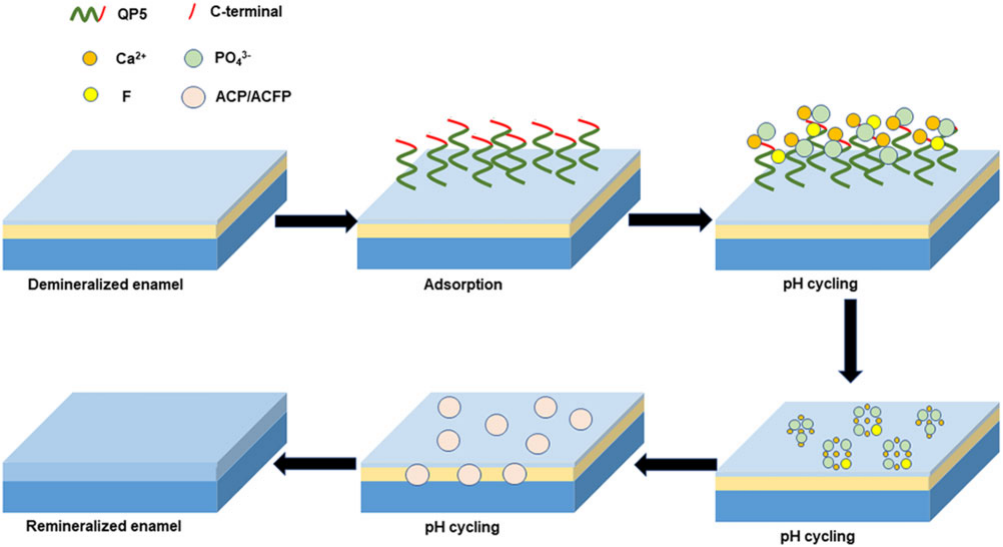
The schematic representation of the enamel remineralization mechanism assisted by QP5 and fluoride

In our study, QP5 had a 20.25 ml/μmol binding affinity for HA and could adsorb on the enamel surface depending on its QPX and C-terminal domain which derived from the structure of amelogenin. Previous studies have shown that acidic groups of proteins or organic molecules can interact with Ca ions to form a calcium complex, and guide calcium phosphate mineralization. Combined with our experimental results, we assumed that QP5 could interact with Ca ions to form a calcium complex relying on acidic amino acid, then, transitorily stabilize the ACP and finally guide it to transform into hydroxyapatite crystals. It could promote calcium and phosphorus redeposition at the enamel surface, at the same time, the QP5–ACP complex could get through the pores access to the lesion interior in demineralized enamel and crystallize. When fluoride ions participated in during remineralization, they incorporated into the apatite structure, forming HA and FHA mixed crystals. We hypothesized that coated peptide at the enamel surface calcium sites would bind fluoride ions, and concomitantly, this biomimetic complex would be capable of inducing mineral deposition. The coated peptide complex would then function as a nucleation site and facilitate the growth of apatite crystals during remineralization. Conversely, the peptide QP5-induced ACP, which could attract fluoride ions to form nanocomplexes that permeated the enamel caries surface to avoid blocking the ion access. When fluoride was used together with casein phosphopeptide, ACP resulted in co-localization of calcium and phosphate ions with fluoride ions at the enamel surface, presumably as amorphous calcium fluoride phosphate (ACFP) nanocomplexes [62]. Our experimental results are consistent with recent studies that support our hypothesis. The hypothetical mechanism of the remineralization process was summarized in Fig. 6: (i) the peptide QP5 absorbed on the enamel surface according to its QPX which could interact with phosphate and calcium on the hydroxyapatite surface; (ii) ACP was formed on the surface of the enamel and QP5 could transitorily stabilize the ACP by interacting with Ca ions in ACP to form the QP5–ACP complex. In the presence of fluoride, the complex could attract fluoride to form QP5–ACFP complex. The complex could get into the deeper layer of carious lesions through the access; (iii) QP5 controlled the formation of oriented hydroxyapatite and fluorohydroxyapatite crystals on/under the demineralized enamel surface.

## Conclusion

This study showed that the biomimetic amelogenin-derived peptide QP5 could bind on the enamel surface, transitorily stabilize the ACP, control the crystallization of hydroxyapatite and promote the remineralization of caries lesions. In the presence of fluoride, it had a positive interaction with fluoride, offering a promising dental material in research into improved fluoride remineralization efficiency. Future studies should further clarify these mechanisms of action and verify its function in animal experiment of enamel caries.

## Funding

This work was supported by the National Natural Science Foundation of China (81470734 and 81771062).

*Conflict of interest statement*. All authors declare no conflict of interest.

## Supplementary Material

rbaa003_Supplementary_Data
