# Supplementary material for: Remineralization of enamel caries by an amelogenin-derived peptide and fluoride in vitro
Source: Regen Biomater. 2020 Mar 3;7(3):283–92. doi: 10.1093/rb/rbaa003 (PMC7266664; doi:10.1093/rb/rbaa003)
Supplement: rbaa003_Supplementary_Data [file rbaa003_supplementary_data.docx]

Supplementary File

Remineralization of Enamel Caries by an Amelogenin-Derived Peptide and Fluoride In Vitro

Longjiang Ding^1^, Sili Han^1^, Kun Wang^1^, Sainan Zheng^1^, Wenyue Zheng^1^, Xiu Peng^1^, Yumei Niu^1^, Wei Li^1^, Linglin Zhang^1*^

^1^State Key Laboratory of Oral Diseases & National Clinical Research Center for Oral Diseases & Department of Cariology and Endodontics, West China Hospital of Stomatology, Sichuan University, Chengdu, China.

^*^Corresponding author: Linglin Zhang, Professor, State Key Laboratory of Oral Diseases & National Clinical Research Center for Oral Diseases & Department of Cariology and Endodontics, West China Hospital of Stomatology, Sichuan University, Chengdu, China. Address: No.14, Section 3 of Renmin Road South, Chengdu, China. Fax: +86-028-85581436; E-mail: zhll_sc@163.com; Tel: +86-028-85503470.


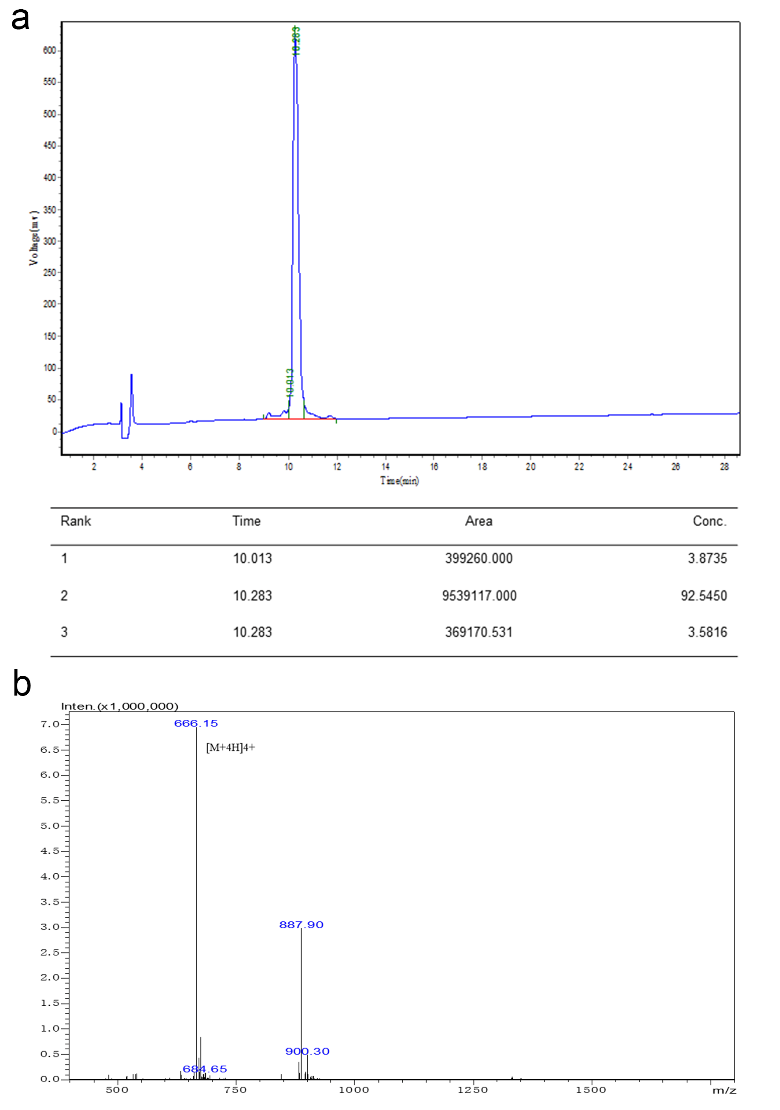


Figure S1. HPLC (a) and mass spectrum (b) analysis of QP5.
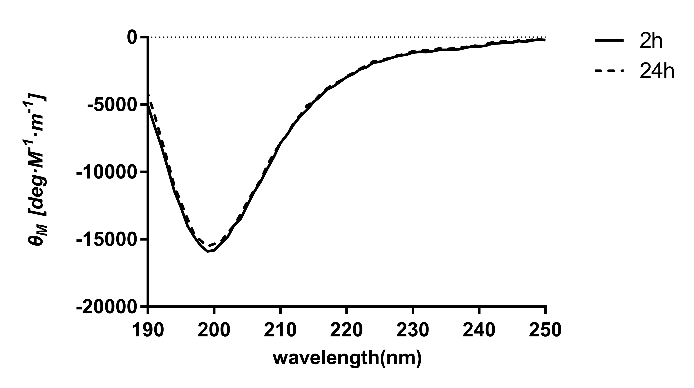


Figure S2. Circular dichroism analysis of QP5. CD spectra of peptide after 2 h (full line) and 24 h (broken line) of incubation.


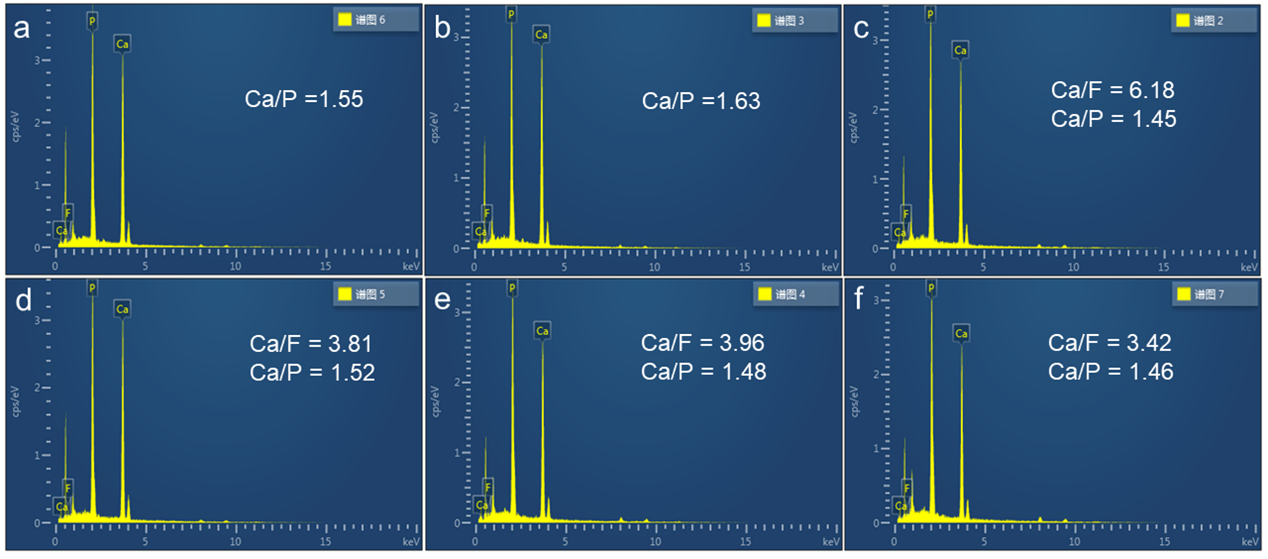


Figure S4. EDXS analysis of enamel surface in all treatment groups. (a) HEPES, (b) QP5, (c) LF, (d)HF, (e) QP5-LF, (f) QP5-HF
